# Report on the 9^th^ International Congress on Peritoneal Surface Malignancies

**DOI:** 10.7497/j.issn.2095-3941.2014.04.008

**Published:** 2014-12

**Authors:** Yan Li, Yang Yu, Yang Liu

**Affiliations:** Hubei Cancer Clinical Study Center, Department of Oncology, Zhongnan Hospital of Wuhan University, Wuhan 430071, China

**Keywords:** Peritoneal surface malignancy, international guidelines, meeting report

The 9^th^ International Congress on Peritoneal Surface Malignancies, sponsored by the Peritoneal Surface Oncology Group International (PSOGI) and organized by the Netherlands Cancer Institute, was held in Amsterdam, the Netherlands, from October 9 to 11, 2014, with over 650 delegates from 66 countries attending the meeting. With the central theme to summarize the global progresses in peritoneal carcinomatosis (PC) diagnosis and treatment, to draft the outline framework of international guidelines on PC treatment, and to formulate the future international collaborative research programs, this congress has set the new historical milestones in the global joint-effort to conquer PC. Major highlights of this congress are reported here.

## The historical background and contemporary significances of recommended guidelines on PC treatment by PSOGI

One of the most significant achievements of this congress is the formulation of the “International Recommendations for cytoreductive surgery (CRS) and hyperthermic intraperitoneal chemotherapy (HIPEC)” or the Amsterdam Statement in short, first drafted by the PSOGI executive committee after serious word-by-word reading and discussion, and then revised and finalized by the congress delegates with new recommendations and corrections.

There is a long and deep historical background behind and certainly will be long-last contemporary impacts ahead of the Amsterdam statement, formulated and proved at this Congress. The Netherlands Cancer Institute at Amsterdam has long been recognized as a world-famous cancer center for PC clinical studies, because the world first phase III prospective randomized clinical trial on CRS + HIPEC to treat PC from colorectal cancer was completed in 2003 at this cancer center by Prof. Vic Verwaal, the president of this congress. From this study, it was found that colorectal PC patients had a median survival of 22.3 months by CRS + HIPEC treatment *vs*. 12.6 months by the standard systemic chemotherapy, a significant improvement in overall survival by 77% (*Journal of Clinical Oncology* 2003;21:3737-3743). Because of encouraging results from this epoch-making study, CRS + HIPEC has been successfully adopted in many European and American countries as standard care for colorectal PC patients, and the current 5-year survival rates for such patients by CRS + HIPEC treatment reach over 50% in the Netherlands, about 25% in the United Kingdom, 30% in France, 35% in Australia and over 30% in the United States.

Another equally important clinical significance of this study is the real breakthrough in colorectal PC treatment. Before the above-mentioned study by Verwaal *et al*., although there had been many treatments for colorectal PC, none of the purely conventional chemotherapy-based therapies could achieve 5-year overall survival, therefore such therapies are in the realm of “palliative care”. By contrast, CRS + HIPEC is the integration of surgical resection of the gross tumor, eradication of residual tumor nodules, micrometastases and free cancer cells by heated chemotherapy and large volume abdominal perfusion washing. This integrated surgical-medical strategy could produce significant synergistic effects to transform some colorectal PC from “incurable disease” to “partially curable disease”, and to change the history from “no long-term survivors” to “numerous long-term survivors”. Therefore CRS + HIPEC could be regarded as a “curative treatment strategy”.

## Key messages from the Amsterdam statement

The Amsterdam statement is the first international guideline for the treatment of PC that outlines the fundamental requirements in performing CRS + HIPEC treatments, such as the indications and contraindications, technical essentials and capabilities of the specialized PC treatment centers, technology transfer and implementation of CRS + HIPEC. The following eight key messages have been outlined in the Amsterdam statement: (I) CRS defined as removal of macroscopic abdominal and peritoneal disease, combined with HIPEC is the treatment indicated for pseudomyxoma peritonei and appendiceal neoplasia with peritoneal metastases; (II) CRS and HIPEC should now be considered as a standard of care for selected patients with peritoneal mesothelioma and moderated to small volume peritoneal metastases secondary to colorectal cancer; (III) patients who have ovarian or advanced peritoneal metastases from gastric cancer may profit from this strategy but additional evidence should be generated from ongoing collaborative studies at experienced treatment centers; (IV) further evidence of neoadjuvant intraperitoneal, systemic chemotherapy and CRS with HIPEC is required for patients with peritoneal metastases from gastric cancer; (V) CRS and HIPEC should be avoided in patients who are unlikely to undergo a complete or near-complete resection, or, due to comorbidities are unlikely to achieve a full recovery; (VI) CRS and HIPEC should not be offered at institutions where there is insufficient knowledge or insufficient skill to (i) achieve a complete cytoreduction and (ii) manage the safe administration of perioperative chemotherapy; (VII) CRS and HIPEC should be offered in experienced centers where the morbidity and mortality is acceptable and the benefit gained by patients far outweighs the risks; (VIII) developing centers should seek support from established teams to assist their development whilst gaining experience in these techniques.

## Prospective randomized clinical trials of prophylactic HIPEC for colorectal cancer patients with high risk of peritoneal metastases

Since CRS + HIPEC has achieved outstanding success in treating colorectal PC patients, the executive committee of PSOGI considers it proper time to conduct well-designed prospective randomized clinical trials of prophylactic HIPEC for colorectal cancer patients with high risk of peritoneal metastases, so as to evaluate how effective of such approach to reduce the risk of peritoneal metastases as well as liver metastases. Colorectal cancer patients with high risk for peritoneal metastases are those with tumors invading the serosa or adjacent structures (stages T_3_/T_4_ by TNM classification), signet-ring cell carcinoma or mucinous adenocarcinoma by histopathology, and ascites during surgical operation.

Dr. Sammartino *et al*. from Sapienza University, Rome, Italy reported the long-term results of prophylactic HIPEC for locoregional control in patients with colonic cancer at high risk of peritoneal metastases. In this controlled clinical trial, advanced colon cancer patients at high risk for peritoneal spread (stages T_3_/T_4_ and mucinous or signet ring cell histology) were divided into the study group (*n*=25) to have curative radical resection plus prophylactic HIPEC treatment, and control group (*n*=50) to have standard curative resection alone. At 48 months after the study closed peritoneal metastases and local recurrences developed significantly less often in the study group (4%) than the control group (28%) (*P*<0.03). Moreover, the median overall survival was also significantly longer in the study group (59.5 months) than the control group (52.0 months) (*P*<0.04). The adverse events rate was similar between the two groups. In addition, multivariate analysis confirmed that prophylactic HIPEC was an independent prognostic factor for improved overall survival and disease-free survival.

Dr. Baratti *et al*. at Fondazione IRCCS Istituto Nazionale dei Tumori, Milano, Italy also reported a matched case-control study to evaluate the efficacy and safety of prophylactic HIEPC in colorectal cancer patients at high risk for developing peritoneal metastases. The study group consisted of 20 patients treated by curative surgery plus HIPEC and the control group consisted of 40 patients treated by the same surgical approach at the same time period. The two groups were well matched for all the baseline clinico-pathological characteristics. By the median follow-up of 5 years, the 5-year cumulative incidence of peritoneal metastases was 5.3% in HIPEC group *vs*. 57. 8% in control group (*P*=0.001). At the time of their reporting, the median overall survival was not reached for the HIPEC group (81.3% of the patients alive at 5 years) *vs*. 66.4 months for the control group (*P*=0.043), and the median progression-free survival was also not reached for the HIPEC group (70.0% of the patients remained progression-free at 5 years) *vs*. 24.8 months for the control group (*P*=0.001). Multivariate analysis also identified HIPEC as an independent factor for reduced PC risk, and improved overall survival and progression-free survival.

These two single-center studies have provided supporting evidence for PSOGI to propose randomized, prospective, multicenter international clinical trials to study the efficacy and safety of prophylactic HIPEC for advanced colorectal cancer patients at high risk of peritoneal metastases.

## CRS + HIPEC to treat PC of ovarian origin

Peritoneal metastasis is the inevitable pathological process for advanced ovarian cancer, and most of the patients with ovarian serous carcinoma have become clinical stage III by the time they seek medical treatment. Ovarian cancer tends to metastasize along peritoneal surface involving the pelvic and abdominal peritoneum including the omentum, surface of the small intestine and colon, mesenterium, peritoneum of colon gutters, diaphragm and the surface of liver and spleen whilst two-thirds of the patients with ascites. These patients could be suitable population for CRS + HIPEC treatment.

There are two case-control studies reported at this Congress demonstrating the advantage of CRS + HIPEC on ovarian cancer. Cascales-Campos from the Virgen De La Arrixaca University Hospital, Murcia, Spain reported a case-control study on 87 patients with stage IIIC/IV ovarian cancer. Of the 87 patients, 52 were treated with HIPEC (paclitaxel 60 mg/m^2^, 60 min, 42 °C) and 35 were control group. The result showed that the 1-year disease-free survival was 81.0% *vs*. 66.0% and 3-year disease-free survival was 63.0% *vs*. 18.0% (*P*<0.05). Multivariate analysis revealed that HIPEC was the independent prognostic factor. In another case-control study by Safra from Israel, 27 recurrent epithelial ovarian cancer patients were treated with CRS + HIPEC and 84 matched-control patients just have surgical resection. The median progression-free survival was 15 months in the HIPEC group and 6 months in the systemic chemotherapy group (*P*<0.01). The 5-year survival rate was significantly higher in the HIPEC treated patients compared to that of the controls (79% *vs*. 45%, *P*<0.05).

More importantly, Dr. Spiliotis *et al*. from Greece conducted a double-blind prospective phase III clinical trial on CRS + HIPEC in patients with recurrent ovarian cancer. The study population included 120 patients with stage IIIC/IV ovarian cancer who experienced disease recurrence after initial surgical treatment and first-line systemic chemotherapy. These patients were randomized into two groups. Group A comprised of 60 patients treated with CRS followed by HIPEC and then systemic chemotherapy. Group B comprised of 60 patients treated with CRS only and systemic chemotherapy. The mean survival was 26.7 months in group A and 13.4 months in group B (*P*<0.01). Three-year survival was 75.0% for group A *vs*. 18.0% for group B (*P*<0.01). In the HIPEC group, the mean survival was not different between patients with platinum-resistant disease *vs*. platinum-sensitive disease (26.6 *vs*. 26.8 months).

## The learning curve and technical training of CRS + HIPEC strategy

Because of the technical complexities and difficulties of CRS + HIPEC procedures, the Congress emphasized the importance of appropriate technical training, and the newly established PC treatment centers should pay serious attention to learning curves, and must receive standardized and structured training to gain essential experience and knowledge for safe and steady development.

Dr. Jimenez *et al*. in Madrid, Spain reported the learning curve of CRS + HIPEC by analysis of 324 PC patients treated over a 13-year period. In the first period from 2000 to 2007, 90 patients were treated; and in the second period from 2008 to 2013, 234 patients were treated. Meticulous analysis of all the treatment variables revealed significantly better clinical outcomes in the second period than the first period, particularly in terms of reduced intraoperative blood transfusion volume, increased completeness of cytoreduction, reduced re-operation rates, reduced intestinal fistula and reduced respiratory complications. It was concluded from this analysis that the learning curve of this procedure is long and the starting centers should perform at least 80 such procedures before they could establish a safe and efficient CRS + HIPEC system.

Prof. Verwaal at the Netherlands Cancer Institute reported his innovative approaches to the learning curve in newly started PC centers. First Dr. Verwaal had a hand-by-hand demonstration and training during the CRS + HIPEC procedures for the first ten operations. Then the trainee surgeons performed such surgery under Dr. Verwaal’s direct supervision at the operation room for another ten operations. After these two periods of direct and indirect training, the trainee surgeons could start their only independent PC program. Analysis of the four centers established under this training model showed the completeness of cytoreduction could be up to 86.0%. In comparison, other centers not trained in this model had only 66.0% of completeness of cytoreduction (*P*<0.001). Meanwhile, the other clinical outcome variables were also significantly better in this new training model.

As European countries have accumulated rich experiences in both clinical studies and technical trainings of CRS + HIPEC, there is increasing need for a unified training program that could be applied throughout Europe. To meet such a demand, Prof. Santiago González-Moreno from M.D. Anderson Cancer Center, Madrid, Spain and Prof. Deraco D from IRCCS Istituto Nazionale dei Tumori, Milano, Italy, two founders of PSOGI, jointly established a European School of Peritoneal Surface Oncology, under the framework of European Society of Surgical Oncologists (ESSO). At the Congress, Prof. Santiago delivered a detailed keynote speech on the program structure, mentors qualification, trainee validation, collaborative networks and technical supporting facilities. This well designed unified and structured training program is now in full operation in Europe, with oncology professionals from UK, France, Germany, Italy, Spain, Sweden, Switzerland, the Netherlands and Greece receiving this structured training.

## Comments and future perspectives

PC is one of the most common forms of locoregional cancer spreading from both gastrointestinal and gynecological malignancies, and has been a long-standing formidable challenge in clinical oncology. In the past, little was known about the disease mechanisms and biological behaviors of PC, and the oncology community in general often regarded PC as terminal stage disease of widespread cancer metastases that deserve only palliative care rather than active treatments, and therefore all the traditional therapies can achieve very limited effect. It is only in recent years that the oncology community recognized PC often behaves as loco-regional spread rather than “widespread metastasis”, thus proactive strategies to control loco-regional diseases could actually bring significant clinical benefits to such patients. After about 30 years of oncology research, CRS + HIPEC as an integrated comprehensive treatment strategy has been developed and proved to be successful treatment for PC.

PSOGI is a collaborative international organization to lead PC clinical treatment, to summarize the major progresses in this field and to direct the future research strategies against peritoneal surface malignancies, with 14 members from 10 countries forming the executive committee. Under the PSOGI lead, 9 international conferences on PC treatment have been successfully held, and significant improvements in PC treatment efficacy have been achieved. The 9^th^ International Congress on Peritoneal Surface Malignancies not only summarized the latest progresses in this field, evaluated the efficacy and safety of CRS + HIPEC for PC from various cancers, but also developed the first ever international guideline recommendations for PC treatment. This Amsterdam statement should promote CRS + HIPEC strategy across the world and help push the PC treatment to another historical new height.

